# A Population Study on COVID-19 Information Sharing: Sociodemographic Differences and Associations with Family Communication Quality and Well-Being in Hong Kong

**DOI:** 10.3390/ijerph19063577

**Published:** 2022-03-17

**Authors:** Shirley Man-Man Sit, Wei-Jie Gong, Sai-Yin Ho, Agnes Yuen-Kwan Lai, Bonny Yee-Man Wong, Man-Ping Wang, Tai-Hing Lam

**Affiliations:** 1 School of Public Health, The University of Hong Kong, Hong Kong, China; shirlsit@hku.hk (S.M.-M.S.); gweijie@connect.hku.hk (W.-J.G.); syho@hku.hk (S.-Y.H.); wongbonny@hotmail.com (B.Y.-M.W.); hrmrlth@hku.hk (T.-H.L.); 2 School of Nursing, The University of Hong Kong, Hong Kong, China; agneslai@hku.hk; 3 School of Nursing and Health Studies, Hong Kong Metropolitan University, Hong Kong, China

**Keywords:** COVID-19, information sharing, information and communication technologies, digital technologies, communication inequalities, family communication, family well-being

## Abstract

Family support through the sharing of information helps to shape and regulate the health and behaviours of family members, but little is known about how families are sharing COVID-19-related information, or about its associations with family communication quality and well-being. We examined the associations of COVID-19 information sharing methods with sociodemographic characteristics, the perceived benefits of information communication and technology (ICT) methods, and family communication quality and well-being in Hong Kong. Of 4852 respondents (53.2% female, 41.1% aged over 55 years), the most common sharing method was instant messaging (82.3%), followed by face-to-face communication (65.7%), phone (25.5%) and social media (15.8%). Female sex (adjusted prevalence ratio (aPR) 1.09), older age (aPRs 1.14–1.22) and higher household income (aPR 1.06) (all *p* ≤ 0.04) were associated with instant messaging use, while post-secondary education was associated with face-to-face (aPR 1.10), video call (aPR 1.79), and email (aPR 2.76) communications (all *p* ≤ 0.03). Each ICT sharing method used was associated with a higher likelihood of both reported benefits (aPRs 1.26 and 1.52), better family communication quality and family well-being (adjusted βs 0.43 and 0.30) (all *p* ≤ 0.001). We have first shown that COVID-19 information sharing in families using both traditional methods and ICTs, and using more types of methods, was associated with perceived benefits and better family communication quality and well-being amidst the pandemic. Sociodemographic differences in COVID-19 information sharing using ICTs were observed. Digital training may help enhance social connections and promote family well-being.

## 1. Introduction

Positive family communication is at the core of a strong and balanced family system, and is the foundation for facilitating the sharing of ideas, feelings and values to maintain and enhance family well-being [1,2]. In times of unpredictable stress, such as the COVID-19 pandemic, direct and supportive communication among family members is crucial to reduce psychological distress and strengthen relationships and functioning amidst widespread social disruption [3]. Support through the sharing of information plays a significant role in shaping and regulating the health and related behaviours of family members [4]. The sharing of health information in families can be defined as a form of distributed health literacy, fostering mutual understanding and support, and is especially vital in contributing to one’s health status, knowledge and behaviours [5]. Family members often rely on each other to seek, share, and interpret health information [6]. However, research and knowledge on how families are sharing COVID-19-related information amidst the pandemic and its associations with family well-being are limited. Our team previously reported that family instant messaging (IM) e-chat group use was associated with greater family well-being and personal happiness [7], and that the frequency of COVID-19 information sharing with family members was associated with preventive behaviours and positive family well-being [8]. Understanding how families are sharing information and the associations with family well-being can inform current and future risk and crisis communication strategies amidst the pandemic and beyond.

We searched PubMed and Cochrane Library using the keywords “COVID-19”, “coronavirus”, “family”, “communication”, “sharing”, “information” and “well-being” up to 3 November 2021. We found one survey which reported the types of sources that people received COVID-19 information from [9], another survey on the perceived trust of COVID-19 information sources in sharing information [10], and our team’s previous survey on the frequency of COVID-19 information sharing with family [8]. We found no survey reports on the different COVID-19 information sharing methods used among family members amidst the pandemic and their associations with perceived benefits and family communication quality and well-being.

Disruptions brought on by the pandemic have steered people away from traditional methods of information sharing such as face-to-face communication and phone calls, towards a growing reliance on newer information and communication technologies (ICTs) such as IM, video calls and social media [11]. Compared with traditional methods, these newer and inexpensive methods possess barrier-breaking functions that allow information to be shared instantaneously and interactively with multiple people at once, regardless of time or location. Sharing via images, videos and audio clips with immediate acknowledgment and feedback increases social connectivity whilst still adhering to social distancing regulations and guidelines. Hence, the use of ICT might have helped some families stay and feel more connected amidst the challenges of the pandemic.

While digital technologies have revolutionized the way we communicate and share information, almost half of the world remain disconnected from the internet, with many people relying on traditional communication avenues such as phone calls from family and friends to receive vital updates on the COVID-19 outbreaks [12]. Hong Kong, one of the most urbanized and westernized cities in China, has widespread internet and ICT penetration, with 94% of households having internet-connected computers at home and 92% of individuals owning smartphones [13]. However, a digital divide remains, primarily affecting older individuals and those of lower socioeconomic status [13]. Our previous reports showed that traditional communication methods such as face-to-face communication and phone calls were still commonly used in Hong Kong families [14,15], but such methods might have changed amidst the pandemic.

We examined the associations of COVID-19 information sharing methods with sociodemographic characteristics, the perceived benefits of ICT, and family communication quality and well-being.

## 2. Materials and Methods

### 2.1. Study Design and Procedures

Under the Hong Kong Jockey Club SMART Family-Link Project, we conducted the Family Amidst COVID-19 (FamCov) survey in May 2020, after the second wave of the pandemic. In anticipation that another outbreak wave could begin any time, we conducted the survey on as large a sample as possible within budget constraints in a short span of six days (from 26 May to 31 May 2020). The target population was Hong Kong residents aged 18 years and above with one or more family members.

The survey design and methods have been published [16,17]. Briefly, 70,984 email invitations were sent to Hong Kong adults with valid email addresses by a well-known local survey agency, the Hong Kong Public Opinion Research Institute. A total of 20,103 email invitations were opened and 4891 respondents who fit the inclusion criteria completed the survey (24.3% response rate). The 39 respondents who did not answer the question on information sharing methods with family were excluded, leaving 4852 for the present analyses. Informed consent was obtained from all respondents before starting the survey. Ethics approval was granted from the Institutional Review Board of the University of Hong Kong/Hospital Authority Hong Kong West Cluster (IRB reference no.: UW20-238).

### 2.2. Measurements

The definition of family (“family members who are related through biological, marital, cohabitation, and/or emotional bonding”) was provided before the questions. COVID-19 information sharing methods with families were assessed by the question, “When the COVID-19 outbreak was severe, what method(s) did you usually use to share COVID-19 information with family members?”. One or more answer options could be selected, which included face-to-face communication, phone, IM, social media (e.g., Facebook, Instagram), video calls, and emails. Our previous paper assessed family IM e-chat group use with the same question format [7].

The perceived benefits of ICT use during COVID-19 were assessed by the question, “What benefits has the use of ICT brought you amidst the pandemic?”. A list of choices of benefits were provided (including “don’t know/refuse to answer” and ”no benefits”), and one or more could be selected. The benefits included in the present analyses focused on family well-being, including strengthening family communication, and improving family relationship. Both perceived benefits were analysed as “yes” vs. “no”. Our previous papers have assessed and reported on the perceived benefits and harms of COVID-19 [16,17].

Family well-being was assessed by three separate questions on family happiness, health and harmony (3Hs), “How happy/healthy/harmonious do you think your family is?” on a scale of 0 (very unhappy/unhealthy/unharmonious) to 10 (very happy/healthy/harmonious), and a composite family well-being score (0 to 10) was calculated by the total 3Hs scores being divided by 3. Family communication quality was assessed by the question, “How do you find the quality of communication between you and your family members?” on a scale of 0 (very poor) to 10 (very good). We reported results based on the above elsewhere [17,18,19,20].

We also collected information on sociodemographic characteristics including sex, age group (18–24, 25–34, 35–44, 45–54, 55–64, and 65 years or above), education (primary or lower, secondary, diploma or certificate, associate degree, and degree or higher), household monthly income (no income, less than HKD 4000, 4000–9999, 10,000–19,999, 20,000–29,999, 30,000–39,999, and 40,000 or higher (USD 1 = HKD 7.8)), housing type (public housing, subsidized housing, and private housing), and whether living with cohabitants (yes vs. no). As in our previous papers [16,17], several variables were recoded for analyses: age (18–24, 25–34, 35–44, 45–54, and 55 years or above), education (secondary or below, and post-secondary), housing type (rented: public housing, subsidized housing, and private housing; and owned: private housing) and household monthly income per person (lower: less than or equal to the median local monthly household income per person; and higher: more than the median).

### 2.3. Statistical Analysis

Characteristics of respondents, presented as number (percentage) for categorical variables and mean ± standard deviation for continuous variables, were weighted by sex, age group, and education levels of the 2019 Hong Kong general population to improve representativeness [21]. Considering that the perceived benefits of ICT use were taken as outcomes, the number of ICT sharing methods was calculated as the sum (range 0 to 5) of the types of ICT sharing methods used, except face-to-face sharing, and was analysed as a continuous or categorical variable (0, 1, 2, and ≥3). Adjusted prevalence ratios (aPRs) with 95% confidence intervals (CIs) were calculated using Poisson regressions with robust variance estimators to examine the associations of sociodemographic characteristics with COVID-19 information sharing methods [22], and adjusted regression coefficients (βs) and 95% CIs were calculated using multivariable linear regressions to examine the association of the number of ICT sharing methods with sociodemographic characteristics, with mutual adjustment. To examine the associations between the perceived benefits of ICT sharing methods, family communication quality, and family well-being, aPRs and adjusted βs with 95% CIs were also calculated, respectively, adjusted for sex, age group, education, housing type, whether living with cohabitants or not, and household monthly income per person. All statistical analyses were performed using Stata 15.1 in Windows. Statistical significance was indicated by a 2-sided *p* < 0.05.

## 3. Results

Table 1 shows that of the 4852 included respondents, after weighting, 53.2% were female, 41.1% were aged 55 years or older, 34.1% had post-secondary education, 63.6% lived in owned housing, 52.5% had lower household monthly income, and 94.6% lived with cohabitants. The most frequent method of sharing COVID-19 information with family was IM (82.3%), followed by face-to-face communication (65.7%), phone (25.5%), social media (15.8%), video calls (5.2%), and emails (2.2%). Respondents used, on average, 1.3 ± 0.9 types of COVID-19 information sharing methods using ICT, and 35.0% used more than one ICT method. A total of 14.3% did not use any ICT methods and only shared information with family face-to-face. More than half (53.1%) reported ICT use benefits of strengthening family communication, and 13.0% reported improving family relationships. Family communication quality had a mean score of 6.6 ± 1.9, and family well-being had a mean score of 7.1 ± 1.6.

Table 2 shows that more females used IM to share COVID-19 information with family (aPR 1.09), but fewer used face-to-face communication (aPR 0.90) and emails (aPR 0.52) (all *p* ≤ 0.05). Older age was positively associated with use of IM (aPRs 1.15–1.23), phone (aPRs 1.65–2.83), and video calls (aPRs 3.29–4.08) (all *p* ≤ 0.04) (all *p* for trend ≤ 0.007), but negatively associated with face-to-face communication (aPRs 0.72–0.88) (all *p* ≤ 0.001) (*p* for trend < 0.001). Post-secondary education was positively associated with face-to-face communication (aPR 1.10), video calls (aPR 1.79), and emails (aPR 2.76) (all *p* ≤ 0.03) but negatively associated with social media (aPR 0.77, *p* = 0.01). Living with cohabitants was positively associated with use of face-to-face communication (aPR 2.30) and social media (aPR 2.06), but negatively associated with IM use (aPR 0.95) (all *p* ≤ 0.03). Respondents with higher household income were associated with IM use (aPR 1.06, *p* = 0.001). Compared with those using no ICT sharing methods, more of those using ≥1 ICT sharing methods were female (aPR 1.06) and of older age groups (aPRs 1.24–1.48) (all *p* ≤ 0.004).

Table 3 shows that sharing COVID-19 information using IM, phone, social media and video calls was associated with both strengthening family communication (aPRs 1.27–1.92) and improving family relationship (aPRs 1.52–3.54) (all *p* ≤ 0.005). All methods, except email, were associated with higher family communication quality (adjusted βs 0.24–0.66), and all methods were associated with better family well-being (adjusted βs 0.17–0.55) (all *p* ≤ 0.04). Using each type of information sharing method was also associated with both perceived benefits (aPRs 1.26 and 1.52) and better family communication quality and well-being (adjusted βs 0.43 and 0.30) (all *p* ≤ 0.001). Those who used no ICT methods and only face-to-face communication to share information had the worst outcomes.

## 4. Discussion

Our study is the first to show that, amidst the pandemic, all COVID-19 information sharing methods (except emails) with family members and using more types of sharing methods were associated with both better family communication quality and family well-being. Our findings also show that IM was the most frequent method of sharing, followed by face-to-face communication. Females, older respondents and those with higher household income were associated with the use of IM.

Our results highlight the increased use of IM as the most frequently used method of COVID-19 information sharing among family members amidst the pandemic, which differs from our findings prior to the pandemic showing face-to-face communication as the most frequently used method to communicate and share information with family members in Hong Kong [14,15]. Although Hong Kong had no lockdowns, fear of infection and social distancing regulations greatly reduced face-to-face communication meetings with family, driving the shift from pre-pandemic in-person communication to spending more time online and using ICTs such as IM and social media [11]. Social and economic disruptions in many countries have also raised the importance of digital technologies in both pandemic response and in meeting the challenges arising in work, education and daily life [23].

Females were associated with the use of IM to share COVID-19 information, which is consistent with our previous pre-pandemic [14,15] and COVID-19 studies [24]. Despite younger people being more familiar with and accustomed to new technologies, we found that more older people used ICTs, including IM and video calls, to share COVID-19 information, but fewer of them used face-to-face communication. As the severity of COVID-19-related complications increases with age, older adults or their family members should adhere more strictly to social distancing regulations. Previous studies suggested that prolonged isolation had incentivised older adults to learn, navigate and embrace new technologies [25,26]. ICTs facilitate social connectivity and can help reduce social isolation, allowing older adults to feel less lonely by increased online interactions and connections with others [27]. Studies have reported the benefits of digital training and adoption in older adults to mitigate the adverse effects of social and spatial barriers and enhance social connections, curb isolation and promote a better quality of life [28,29,30]. We must not underestimate that self-efficacy and motivation, especially when learning and using ICTs, can bring in many immediate benefits, in addition to family communication and well-being.

More respondents with higher education shared COVID-19 information via face-to-face communication and video calls, but fewer shared information via social media. Social media is a crucial communication tool that provides direct and free access to unlimited information that may or may not be credible, possibly contributing to the ongoing infodemic [31]. Those with higher education may be more cautious or sceptical about the reliability of information presented on such platforms and be less inclined to share with family. Consistent with previous studies, higher household income was associated with IM use [14]. This is expected, as these individuals may have more access to ICTs. Around 97% of Hong Kong families with a monthly household income of HKD 50,000 or more had internet-connected computers at home, which was much higher than that of 34% among families with a monthly household income of less than HKD 10,000 [13]. Our results suggest that the less privileged group would need more help to reduce the impacts of the infodemic on them and their families.

While our previous pre-pandemic study found that traditional methods of communication, including face-to-face communication and phone, were associated with higher levels of family well-being [14], the present study provides new evidence that COVID-19 information sharing methods, both traditional and emerging ICTs such as IM, phone, social media and video calls, were associated with better family communication quality and family well-being. The one exception was email, which was the only method not associated with higher family communication quality. While emails may be utilized effectively in clinical settings between patients and providers or in work settings [32,33], its rigid and professional nature may hinder communication quality within families. Email incivility, or rudeness through email communication, is also common and can have negative impacts [33]. Our null result suggests that our positive results on other sharing methods were unlikely due to social desirability bias and indicates that email is not a preferred method of family communication.

More remarkably, we found that those using more types of information sharing methods were more likely to perceive ICT as beneficial to family communication and relationships, and showed better family communication quality and well-being. Some respondents could have chosen different methods under different contexts to communicate with different family members. Our results suggest that these people had better communication skills and a stronger desire to communicate and share information, and hence perceived more benefits of using ICT. Alternatively, those who did not use different types of methods, specifically no ICT methods, showed the worst outcomes. Our recent paper examining the associations of face-to-face communication and instant messaging family communication with family well-being amidst the pandemic also concluded that individuals who communicated with family using only face-to-face communication had lower personal happiness, family well-being and family communication quality [34]. The widespread use of digital technologies to share information, along with its perceived benefits, is useful in informing the development of tailored risk and crisis communication strategies and strengthening pandemic management [35].

Moreover, as families are an important source of health information, the sharing of such information can be viewed as a form of support and collaboration in helping build a culture of health within the family [36]. For many older adults, seeking and accessing health information, especially digitally, can be challenging. In turn, they often utilize family members to gather, interpret and share important health information [6]. Amidst the challenges and uncertainties of the pandemic, receiving and sending health information to and from family members can help them to develop and maintain healthy attitudes and behaviours, and would also contribute to a team mentality and solidarity, alongside fostering connectedness and family resilience.

With the post-pandemic surge in digitalization and reliance on digital technologies to communicate, additional efforts are needed to improve equitable access and reduce barriers in technology adoption among different groups and communities, especially the underserved or marginalized communities that do not have or have limited access to ICTs, to reduce the digital divide [27]. With the pandemic exposing the gap in access to digital technologies, the United Nations has emphasized the importance of closing the digital divide to foster social inclusion and digital equality [37]. The World Health Organization has also mobilized global stakeholders and resources to reduce communication inequalities at both macro and individual levels to promote health and well-being [38,39]. Our results on the associations, if causal, can add new knowledge to support the development of interventions.

Our study had a few limitations. First, causal relationships cannot be inferred from this cross-sectional survey. Second, all outcomes were self-reported and might be subject to recall and response biases. Third, we only asked respondents whether they lived with cohabitants, and not specifically family members, which might have influenced the methods by which they shared information with family. Fourth, we did not ask for details about what kind of COVID-19 information was shared, and future studies should further investigate the types and contents of information shared to aid health promotion and pandemic response. Lastly, the generalizability of our results could be limited as the online survey undersampled those who were older and had lower education and income, and the contexts of the pandemic were different in different regions. However, the results of the key variables when unweighted and weighted were similar.

## 5. Conclusions

We have first shown that COVID-19 information sharing methods in families, both traditional and using ICTs, and using more types of methods, were associated with perceived benefits and better family communication quality and well-being amidst the pandemic. IM was the most commonly used method of sharing COVID-19 information in families, highlighting the growing importance and reliance on digital technologies in meeting post-pandemic challenges and improving family communication. Sociodemographic differences in COVID-19 information sharing using ICTs were observed. Digital training may help enhance social connections and promote family communication and well-being. Equitable access to and literacy in ICTs are needed to reduce the digital divide.

## Figures and Tables

**Table 1:** Characteristics of the survey sample (*n* = 4852).

	Unweighted ^a^ *n* (%)	Weighted ^b^ *n* (%)
	Total	
Sociodemographics		
Sex		
Male	2111 (43.5)	2259 (46.8)
Female	2741 (56.5)	2568 (53.2)
Age group, years		
18–24	211 (4.4)	405 (8.4)
25–34	1080 (22.3)	745 (15.4)
35–44	1348 (27.8)	818 (16.9)
45–54	1198 (24.7)	875 (18.1)
≥55	1015 (20.9)	1984 (41.1)
Education		
Secondary or below	654 (13.6)	3160 (65.9)
Post-secondary	4165 (86.4)	1634 (34.1)
Housing type		
Rented	1586 (33.9)	1718 (36.4)
Owned	3100 (66.1)	3002 (63.6)
Household monthly income per person ^c^		
Lower	1254 (29.7)	2172 (52.5)
Higher	2965 (70.3)	1967 (47.5)
Living with cohabitants		
Yes	4505 (94.5)	4498 (94.6)
No	263 (5.5)	255 (5.4)
Methods of COVID-19 information sharing with family		
Instant messaging (Yes)	4066 (83.8)	3973 (82.3)
Face-to-face (Yes)	3321 (68.5)	3169 (65.7)
Phone (Yes)	1184 (24.4)	1229 (25.5)
Social media (e.g., Facebook, Instagram) (Yes)	760 (15.7)	765 (15.8)
Video calls (Yes)	262 (5.4)	250 (5.2)
Emails (Yes)	71 (1.5)	109 (2.2)
Number of ICT sharing methods (Mean ± SD) ^d^	1.3 ± 0.8	1.3 ± 0.9
Number of ICT sharing methods		
0	638 (13.2)	690 (14.3)
1	2528 (52.1)	2451 (50.8)
2	1311 (27)	1263 (26.2)
≥3	375 (7.7)	423 (8.8)
Perceived ICT benefits on family		
Strengthening family communication (Yes)	2404 (51.2)	2459 (53.1)
Improving family relationship (Yes)	527 (11.2)	603 (13.0)
Family outcomes, Mean ± SD ^e^		
Family communication quality	6.5 ± 2.0	6.6 ± 1.9
Family well-being ^f^	7.0 ± 1.7	7.1 ± 1.6

^a^ Missing data were excluded. ^b^ Weighted by sex, age, and education of the 2019 Hong Kong population. ^c^ Income were divided by household size and dichotomized into “lower” (less than or equal to median monthly household income) and “higher”. ^d^ Range 0 to 5, face-to-face sharing was excluded. ^e^ Scale of 0 to 10, with higher scores indicating better outcomes. ^f^ Sum of scores of family happiness, health and harmony, divided by 3.

**Table 2:** Associations of sociodemographic characteristics with COVID-19 information sharing methods ^a^ (*n* = 4852).

	COVID-19 Information Sharing Methods, aPR (95% CI)						Number of ICT Sharing Methods ^b^, Adjusted β (95% CI)	Using ≥1 ICT Method (vs. Using 0 ICT Method), Adjusted aPR (95% CI)
	Instant Messaging	Face-to-Face	Phone	Social Media	Video Calls	Emails		
Sex (vs. Male)								
Female	1.09 (1.06, 1.12) ***	0.90 (0.87, 0.94) ***	1.02 (0.92, 1.14)	0.99 (0.86, 1.14)	1.20 (0.92, 1.55)	0.52 (0.30, 0.88) *	0.08 (0.02, 0.13) **	1.06 (1.02, 1.10) **
Age group, years (vs. 18–24)								
25–34	1.15 (1.03, 1.28) *	0.88 (0.82, 0.94) ***	1.65 (1.03, 2.63) *	1.34 (0.85, 2.09)	2.05 (0.64, 6.56)	−	0.22 (0.09, 0.36) **	1.24 (1.09, 1.41) **
35–44	1.23 (1.11, 1.37) ***	0.72 (0.67, 0.78) ***	2.47 (1.56, 3.91) ***	1.79 (1.16, 2.77) **	3.65 (1.16, 11.45) *	0.23 (0.02, 2.62)	0.45 (0.32, 0.59) ***	1.48 (1.3, 1.68) ***
45–54	1.22 (1.10, 1.36) ***	0.74 (0.69, 0.80) ***	2.83 (1.78, 4.48) ***	1.28 (0.81, 2.00)	3.29 (1.04, 10.47) *	2.11 (0.27, 16.39)	0.43 (0.29, 0.57) ***	1.46 (1.28, 1.66) ***
≥55	1.23 (1.10, 1.37) ***	0.77 (0.71, 0.83) ***	2.48 (1.56, 3.95) ***	0.81 (0.51, 1.30)	4.08 (1.28, 13.03) *	6.35 (0.86, 46.66)	0.39 (0.25, 0.53) ***	1.41 (1.24, 1.61) ***
*p* for trend	<0.001	<0.001	<0.001	0.35	0.007	<0.001	<0.001	
Education (vs. Secondary or below)								
Post-secondary	1.01 (0.97, 1.05)	1.10 (1.02, 1.18) *	1.04 (0.89, 1.22)	0.77 (0.63, 0.94) *	1.79 (1.12, 2.84) *	2.76 (1.12, 6.81) *	0.02 (−0.05, 0.10)	1.02 (0.96, 1.08)
Housing type (vs. Rented)								
Owned	0.98 (0.96, 1.01)	1.01 (0.97, 1.06)	0.92 (0.82, 1.03)	0.95 (0.82, 1.10)	0.98 (0.73, 1.30)	2.19 (1.00, 4.78)	−0.04 (−0.09, 0.02)	0.97 (0.93, 1.01)
Living with cohabitants (vs. No)								
Yes	0.95 (0.91, 1.00) *	2.30 (1.89, 2.82) ***	0.91 (0.74, 1.12)	2.06 (1.32, 3.23) **	1.78 (0.88, 3.60)	1.26 (0.41, 3.93)	0.04 (−0.07, 0.15)	1.04 (0.99, 1.09)
Household monthly income per person (vs. Lower)								
Higher	1.06 (1.03, 1.10) **	1.00 (0.95, 1.04)	1.01 (0.89, 1.14)	1.05 (0.89, 1.24)	0.84 (0.63, 1.12)	1.28 (0.71, 2.33)	0.05 (−0.01, 0.11)	1.03 (0.97, 1.10)

aPR: adjusted prevalence ratio; CI: confidence interval; aOR: adjusted odds ratio. * *p* < 0.05, ** *p* < 0.01, *** *p* < 0.001. ^a^ Mutually adjusted for each other. ^b^ Face-to-face sharing was excluded, considering that the outcomes were the perceived benefits of ICT use.

**Table 3:** Associations of COVID-19 information sharing methods with perceived benefits of ICT and family communication quality and well-being ^a^.

	Strengthening Family Communication (Yes vs. No), aPR (95% CI)	Improving Family Relationship (Yes vs. No), aPR (95% CI)	Family Communication Quality ^c^, Adjusted β (95% CI)	Family Well-Being ^c^, Adjusted β (95% CI)
Methods of COVID-19 information sharing with family (yes vs. no)				
Instant messaging	1.92 (1.69, 2.18) ***	3.54 (2.32, 5.41) ***	0.53 (0.37, 0.69) ***	0.41 (0.27, 0.54) ***
Face-to-face	0.94 (0.89, 1.01)	1.00 (0.83, 1.21)	0.24 (0.11, 0.37) ***	0.17 (0.06, 0.28) **
Phone	1.27 (1.20, 1.35) ***	1.52 (1.27, 1.82) ***	0.58 (0.45, 0.72) ***	0.42 (0.30, 0.53) ***
Social media (e.g., Facebook, Instagram)	1.31 (1.22, 1.40) ***	1.73 (1.42, 2.11) ***	0.54 (0.38, 0.70) ***	0.34 (0.21, 0.48) ***
Video calls	1.36 (1.25, 1.49) ***	1.89 (1.45, 2.47) ***	0.66 (0.40, 0.92) ***	0.55 (0.33, 0.76) ***
Emails	1.11 (0.93, 1.33)	1.85 (1.21, 2.84) **	0.46 (−0.05, 0.96)	0.44 (0.02, 0.87) *
Number of ICT sharing methods ^b^				
Continuous (0–5)	1.26 (1.22, 1.30) ***	1.52 (1.41, 1.65) ***	0.43 (0.36, 0.50) ***	0.30 (0.25, 0.36) ***
≥3 as reference group				
0	0.36 (0.30, 0.42) ***	0.11 (0.06, 0.20) ***	−1.26 (−1.52, −1.00) ***	−0.95 (−1.17, −0.73) ***
1	0.70 (0.64, 0.75) ***	0.45 (0.35, 0.57) ***	−0.84 (−1.06, −0.62) ***	−0.62 (−0.81, −0.44) ***
2	0.83 (0.77, 0.90) ***	0.66 (0.52, 0.84) **	−0.29 (−0.52, −0.05) *	−0.27 (−0.47, −0.07) **
0 as reference group				
1	1.95 (1.67, 2.28) ***	4.03 (2.27, 7.16) ***	0.42 (0.24, 0.60) ***	0.32 (0.17, 0.47) ***
2	2.34 (1.99, 2.74) ***	5.89 (3.30, 10.52) ***	0.98 (0.78, 1.18) ***	0.68 (0.51, 0.84) ***
≥3	2.80 (2.38, 3.31) ***	8.97 (4.96, 16.25) ***	1.26 (1.00, 1.52) ***	0.95 (0.73, 1.17) ***
*p* for trend	<0.001	<0.001	<0.001	<0.001

aPR: adjusted prevalence ratio; CI: confidence interval; * *p* < 0.05, ** *p* < 0.01, *** *p* < 0.001. ^a^ Adjusted for sex, age group, education, housing type, whether living with cohabitants or not, and household monthly income per person. ^b^ Face-to-face sharing was excluded, considering that the outcomes were the perceived benefits of ICT use. ^c^ Scale of 0 to 10, with higher scores indicating better outcome.

## Data Availability

The data presented in this study are available on request from the corresponding authors. The data are not publicly available because our analyses and paper writing on the results are in progress.
